# Impact of CD200-Fc on dendritic cells in lupus-prone NZB/WF1 mice

**DOI:** 10.1038/srep31874

**Published:** 2016-08-22

**Authors:** Yufeng Yin, Lidan Zhao, Fengchun Zhang, Xuan Zhang

**Affiliations:** 1 Department of Rheumatology & Clinical Immunology, Key Laboratory of Rheumatology & Clinical Immunology, Ministry of Education, Peking Union Medical College Hospital; Clinical Immunology Center, Chinese Academy of Medical Science, Beijing, 100730 China

## Abstract

Abnormal expression of CD200/CD200R1 may contribute to the immunologic abnormalities in patients with systemic lupus erythematosus (SLE). This study aimed to assess the function of CD200/CD200R1and impact of CD200-Fc on dendritic cells in lupus-prone NZB/WF1 mice. Female NZB/WF1 mice were treated with CD200-Fc or control for 4 weeks. Plasma samples were collected to measure autoantibody levels. The expression levels of CD200/CD200R1 in peripheral blood mononuclear cells (PBMCs) and splenocytes were examined. The percentage of CD200/CD200R1-positive cells in splenocytes from NZB/WF1 mice was lower than that of C57BL/6 mice (p < 0.05). The plasma level of anti-dsDNA was significantly higher in NZB/WF1 mice than C57BL/6 mice (p < 0.001). However, the anti-dsDNA levels decreased (p = 0.047) after CD200-Fc treatment. Finally, CD200-Fc reduced the levels of IL-6 (p = 0.017) and IL-10 (p = 0.03) in the dendritic cell culture supernatant. This study suggests that the immunosuppressive CD200/CD200R1 signaling pathway might be involved in the immunopathology of NZB/WF1 mice; the present results merit further exploration of agents that can modulate the CD200/CD200FR1 pathway as a therapy for human lupus.

Systemic lupus erythematosus (SLE) is a prototypic autoimmune disease characterized by chronic systemic inflammation accompanied by multi-organ system involvement^1^. Previous studies have shown that more than 100 types of autoantibodies, including anti-dsDNA antibody, anti-Sm and anti-nuclear antibodies(ANAs),are detected in the serum of patients with SLE^2^.

Insufficient clearance of apoptotic material by dendritic cells (DCs), which leads to the release of modified autoantigens that can initiate an immune response, has been thought to play a pivotal role in the immunopathogenesis of SLE^3^. DCs recognize and process antigens for presentation to T cells^4^, and mouse models demonstrated that depletion of DCs results in the disruption of the self-tolerance of CD4^+^ T cells and finally development of spontaneous autoimmunity^5^. Phagocytosis of apoptotic material leads to the maturation of myeloid dendritic cells (mDCs) and production of proinflammatory cytokines, including IL-6^6^. In the presence of IL-6 and other proinflammatory cytokines, mature mDCs can induce the activation of Th1, Th2 and Th17 cells, whereas IL-6 inhibits the development and activity of regulatory T cells (Tregs) in the initiation phase of SLE^7^. Plasmacytoid dendritic cells (pDCs), on the other hand, preferentially play a phagocytic role in SLE progression and lead to increasing concentrations of immune complexes and local inflammation^3^.

CD200, a type I transmembrane glycoprotein, is broadly expressed in diverse cell types ranging from lymphocytes and follicular DCs to central nervous system (CNS) neurons^8^. CD200R1 has been reported to be expressed mainly on cells of myeloid lineage, including macrophages, DCs, and neutrophils^9^,^10^,^11^. As an inhibitory signal pathway, a lack of CD200 or CD200R1, which is the receptor with the highest binding affinity to CD200, resulted in a more rapid onset of experimental autoimmune encephalomyelitis (EAE), increased susceptibility to collagen-induced arthritis (CIA) and aggravation of experimental autoimmune uveoretinitis (EAU) in mouse models^12^,^13^. Meanwhile, CD200-Fc ameliorated the inflammatory changes in these models and targeted proinflammatory cytokine expression^14^,^15^.

However, the role of the CD200/CD200R1 pathway in SLE remains unknown. Our previous study demonstrated that the expression of CD200 and CD200R1 is higher and lower, respectively, in DCs, including both pDCs and mDCs, in patients with SLE compared with healthy controls, which may contribute to immunologic abnormalities and can be corrected by CD200-Fc treatment through reducing DC migration in patients with SLE^16^. In addition, the possible therapeutic potential of targeting the CD200/CD200R pathway with CD200Fc was not fully investigated in SLE. In this study, we aimed to determine the expression of CD200/CD200R1 on peripheral blood mononuclear cells (PBMCs) and subtypes of DCs and to treat lupus-prone NZB/WF1 mice with intraperitoneal injections of recombinant CD200-Fc proteins to investigate the effects of intervening with the CD200 pathway in SLE.

## Materials and Methods

### Mice and treatment

Female C57BL/6 and NZB/WF1 mice were purchased from Weitonglihua (Beijing, China) and the Jackson Laboratory (Bar Harbor, ME, USA), respectively, and were used at 20 to 22 weeks of age. All mice were housed under specific pathogen-free (SPF) conditions. All procedures were performed according to the National Institutes of Health Guide for Care and Use of Laboratory Animals and were approved by the Institutional Animal Care and Use Committee at the Peking Union Medical College Hospital, Chinese Academy of Medical Sciences.

All mice were divided into the following three groups: 1) NZB/WF1 group: five female NZB/WF1 mice were intraperitoneally injected with normal saline (1 mL/mouse); 2) CD200-Fc group: five NZB/WF1 mice were intraperitoneally injected with CD200-Fc recombinant protein (10 μg/mouse); 3) five age- and sex-matched C57BL/6 mice were defined as the controls and were intraperitoneally injected with normal saline (1 mL/mouse). All mice were treated for 4 weeks at 2-day intervals, and samples of spleen and peripheral blood were collected after treatment.

### Preparation of spleen cells and cell separation

Single spleen suspensions were prepared aseptically and re-suspended at 5 × 10^6^ cells/mL in RPMI 1640 supplemented with fetal calf serum (10%), L-glutamine (2 mM), penicillin/streptomycin (100 U/mL), sodium pyruvate (1 mM), HEPES (10 mM), and 2-mercaptoethanol (50 mM). Red blood cells were lysed using BD Pharm Lyse (BD Bioscience, San Jose, CA). Pan dendritic cells (Pan DCs) were isolated using CD11c^+^ and Anti-mPDCA-1^+^ Pan Dendritic Cell MicroBeads (MiltenyiBiotec, BergischGladbach, Germany), cultured at 37 °C in a 5% CO_2_ incubator for the indicated time periods, and activated with rmGM-CSF (100 ng/mL, PeproTech, Rocky Hill, USA) and rmIL-4 (100 ng/mL, PeproTech, Rocky Hill, USA) for 24 hours.

### Flow cytometric analysis (FCM)

Venous blood was collected from the orbital sinus of each mouse after anesthesia, and PBMCs were prepared by Ficoll-Hypaque density-gradient centrifugation. Cell-surface antigen expression on the splenocytes and PBMCs were determined using mAbs purchased from eBioscience (San Diego, CA, USA) unless otherwise stated; the mAbs included CD200 (OX90.1), CD11c (N418), CD11b (M1/70),CD45R (B220) (RA3-6B2), and CD200R1 (OX110) (R&D system, (Minneapolis, MN,USA)and were either unlabeled or labeled with phycoerythrin, fluorescein isothiocyanate, or allophycocyanin. In all experiments, negative isotype control mAbs of the respective IgGs were included to allow accurate background limits, and all incubations were performed and maintained at 4 °C.

### Enzyme-linked immunosorbent assay (ELISA)

The ELISA assay was performed to detect the level of anti-nuclear antibody (ANA), anti-dsDNA and anti-RNP/Sm antibodies (Alpha Diagnostic International, San Antonio, TX, USA) in murine plasma diluted at 1:100. The supernatants of cultured Pan DCs were pooled from replicate wells and subjected to ELISA assays to detect the production of cytokines, including IL-6, IL-10, IL-12 and TNF-α (eBioscience, San Diego, CA, USA). All detections were performed according to the manufacturer’s instructions.

### Histologic assessment of kidneys and urinary protein assays

The kidneys of the mice were harvested, fixed in 10% neutral-buffered formalin, paraffin embedded and cut into 3.5-μm-thick sections. All sections were stained with Periodic Acid-Schiff (PAS). The glomerular pathology was scored based on the previous study as follows: 0, normal (35–40 cells/gcs); 1, few lesions with slight proliferative changes and mild hypercellularity (41–50 cells/gcs); 2, moderate hypercellularity (51–60 cells/gcs), segmental and/or diffuse proliferative changes, hyalinosis, and moderate exudates; and 3, severe hypercellularity (>60 cells/gcs) with segmental or global sclerosis and/or severe necrosis, crescent formation, and heavy exudation^17^. Finally, urinary protein was determined using Multistix (Bayer-Siemens, Erlangen, Germany) and a urine analyzer.

### Statistical analysis

All data were analyzed using SPSS 21.0 software (SPSS Inc., Chicago, IL, USA). An independent-sample t test was used to compare the differences between the groups or differences before and after treatment. P-values < 0.05 were considered statistically significant.

## Results

### CD200 and CD200R1 expression in lupus-prone NZB/WF1 and C57BL/6 mice

**Figure 1:**
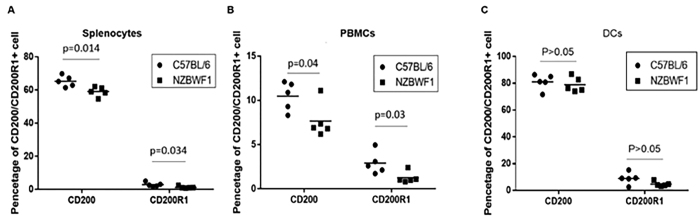
The expression of CD200 and CD2001 in splenocytes, peripheral blood mononuclear cells (PBMCs) and separated dendritic cells (DCs) of C57BL/6 and NZB/WF1 mice. (**A**) CD200 and CD200R1 expression in splenocytes of C57BL/6 and NZB/WF1 mice. (**B**) The percentages of CD200 and CD200R1+ cells in the PBMCs of C57BL/6 and NZB/WF1 mice. (**C**) The percentages of CD200 and CD200R1+ cells in DCs of C57BL/6 and NZB/WF1 mice. The means are depicted as solid lines.

We first determined the percentages of CD200^+^ and CD200R1^+^ cells in splenocytes and peripheral blood cells in the NZB/WF1 and C57BL/6 mice. The proportions of CD200^+^ and CD200R1^+^ cells in splenocytes of NZB/WF1 mice were lower than that of the C57BL/6 mice (59.07 ± 2.92% vs. 65.31 ± 3.33%, p = 0.014 and 2.56 ± 0.52% vs. 4.42 ± 0.98%, p = 0.034, respectively) (Fig. 1A). Additionally, the percentages of CD200^+^ and CD200R1^+^ cells in the peripheral blood mononuclear cells (PBMCs) of the NZB/WF1 mice were lower than that of the C57BL/6 mice (7.66 ± 1.96% vs. 10.48 ± 1.63%, p = 0.04 and 1.26 ± 0.65% vs. 2.90 ± 1.29%, p = 0.03, respectively) (Fig. 1B).In contrast, the proportion of CD200^+^ and CD200R1^+^ cells in DCs did not differ between the NZB/WF1 and C57BL/6 mice (81.01 ± 5.76% vs. 78.25 ± 5.14%, p > 0.05 and 8.96 ± 4.53% vs. 4.70 ± 1.78%, p > 0.05, respectively) (Fig. 1C).

### Effects of CD200-Fc on SLE pathology

**Figure 2:**
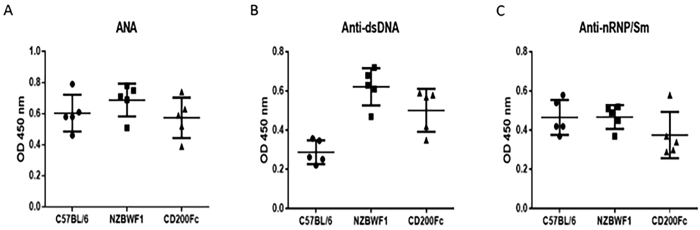
Comparisons of the levels of antinuclear antibody (ANA) and of the anti-dsDNA and anti-nRNP/Sm antibodies in the plasma of NZB/WF1 and C57BL/6 mice. The means and interquartile ranges are depicted as solid lines.

We detected the plasma levels of antinuclear antibody (ANA) and of the anti-dsDNA and anti-nRNP/Sm antibodies using ELISA methods after treatment for 4 weeks. Our findings demonstrated that the OD450 value for anti-dsDNA was significantly higher in the NZB/WF1 group compared with the C57BL/6 group (0.62 ± 0.10 vs. 0.29 ± 0.06, p < 0.001), while there was no significant difference between the levels of ANA and anti-nRNP/Sm (0.69 ± 0.11 vs. 0.60 ± 0.12, p > 0.05 and 0.43 ± 0.05 vs. 0.47 ± 0.09, p > 0.05, respectively) between these two groups. In the CD200-Fc group, the anti-dsDNA level was significantly lower than that of the NZB/WF1 group (0.48 ± 0.10 vs. 0.62 ± 0.10, p = 0.047) (Fig. 2).

**Figure 3:**
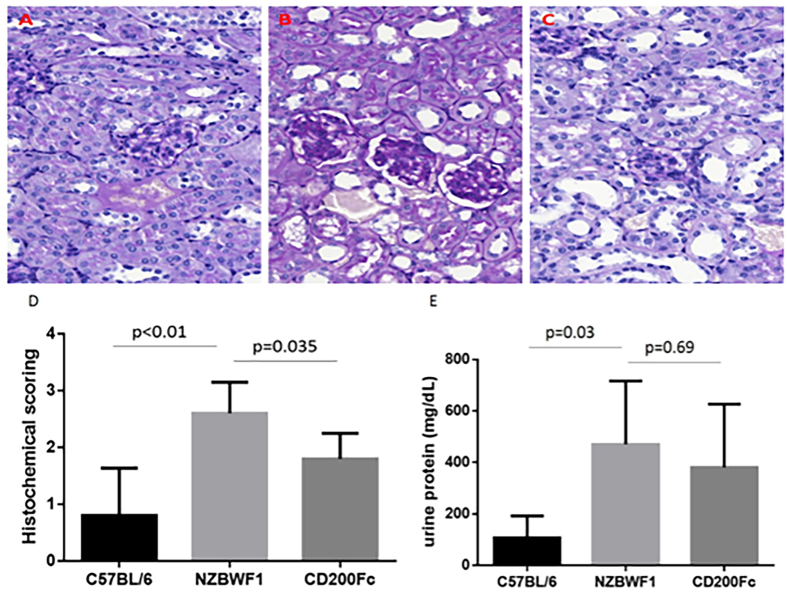
The renal pathology and urine protein of C57BL/6, NZB/WF1 and NZB/WF1 mice treated with CD200-Fc. (**A**) A renal section of C57BL/6 mice treated with normal saline showed the common histological characteristics. (**B**) A kidney from an NZB/WF1 mouse exhibited glomerular hypercellularity and proliferative glomerulonephritis. (**C**) The histopathology of proliferative glomerulonephritis in NZB/WF1 mice was ameliorated with CD200-Fc therapy. (**D**) PAS histopathological scoring of the kidney of C57BL/6, NZB/WF1 and NZB/WF1 mice treated with CD200-Fc. (**E**) The level of urine protein in these three groups of mice. Periodic acid-Schiff (PAS) staining; magnification, x 400.

We next determined the effect of CD200-Fc on renal pathology and the level of urine protein in NZB/WF1 mice. The kidneys of C57BL/6 mice treated with normal saline showed common histological characteristics (Fig. 3A), while the kidney from an NZB/WF1 mouse exhibited glomerular hypercellularity, proliferative glomerulonephritis and a higher PAS score compared with the C57BL/6 mice (5.00 ± 2.60 vs. 0.80 ± 0.84, p < 0.01) (Fig. 3B,D). For the CD200-Fc group, the histopathology of proliferative glomerulonephritis was ameliorated with lower PAS scores (1.80 ± 0.45 vs. 5.00 ± 2.60, p = 0.035) (Fig. 3C,D). The urine protein of the NZB/WF1 group was higher than that of the C57BL/6 group (470.0 ± 246.5 mg/dL vs. 107.0 ± 84.9 mg/dL, p = 0.03), and the urine protein between the CD200-Fc and NZB/WF1 groups was not significantly different but there appeared to be a decreasing trend (380.00 ± 246.5 mg/dL vs. 470.00 ± 246.5 mg/dL, p = 0.69) (Fig. 3E).

### Effects of CD200-Fc on DCs in lupus-prone NZB/WF1 mice

**Figure 4:**
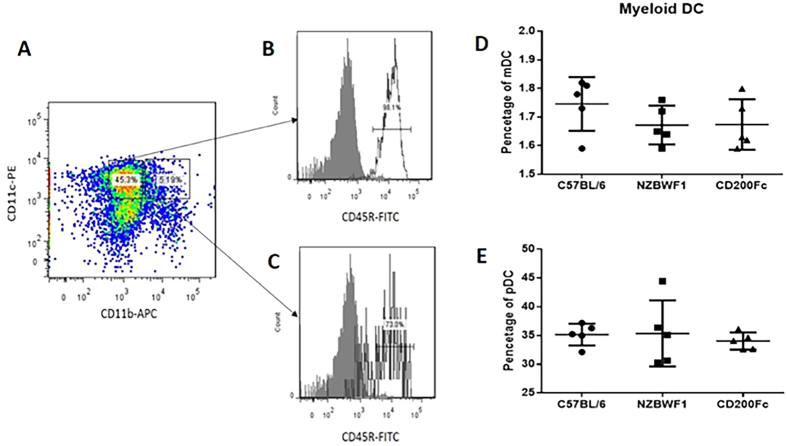
The percentage of two subsets of dendritic cells in the splenocytes of NZB/WF1 and C57BL/6 mice. (**A**,**B**,**C**) Gating of CD11b^+^CD11c^high^CD45R^−^ mDCs and CD11b^-^CD11c^interm.^CD45R^+^ pDCs. Open histogram: mAb against CD45R; gray histogram: IgG control. (**D**) Percentages of mDCs in splenocytes of C57BL/6 and NZB/WF1 mice and of NZB/WF1 mice treated with CD200-Fc. (**E**) Percentages of pDCs in splenocytes of C57BL/6 and NZB/WF1 mice and of NZB/WF1 mice treated with CD200-Fc. The means and interquartile ranges are depicted as solid lines.

We first examined the expression of CD200 and CD200R1 in subsets of DCs, including mDCs (CD11b^+^CD11c^high^CD45R^−^) and pDCs (CD11b^−^CD11c^interm.^CD45R^+^). The results demonstrated that there were no differences between the percentages of the mDCs and pDCs in NZB/WF1 and C57BL/6 mice (1.67 ± 0.03% vs. 1.75 ± 0.04%, p = 0.19 and 35.37 ± 5.73% vs. 35.17 ± 1.91%, p = 0.94, respectively). In the CD200-Fc group, the mDC and pDC subsets were not significantly different from those of the NZB/WF1 group (1.68 ± 0.09% vs. 1.67 ± 0.03%, p = 0.97 and 34.04 ± 1.48% vs. 35.37 ± 5.73%, p = 0.63, respectively) (Fig. 4).

**Figure 5:**
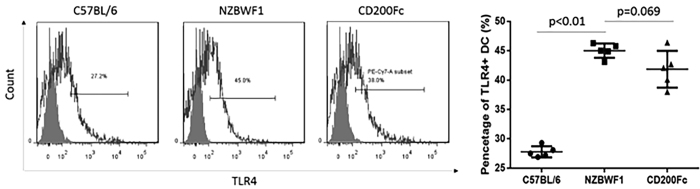
Expression of Toll-Like Receptor (TLR4) in DCs from C57BL/6 and NZB/WF1 mice and from NZB/WF1 mice treated with CD200-Fc. The means and interquartile ranges are depicted as solid lines.

Next, we detected the expression of Toll-Like Receptor 4 (TLR4) on the surface of DCs. As shown in Fig. 5, the expression of TLR4 was significantly higher on the DCs of NZB/WF1 compared with C57BL/6 mice (45.02 ± 1.22% vs. 27.80 ± 0.95%, p < 0.01). However, there was no significant difference in the TLR4 expression on the DCs of the CD200-Fc group compared with the NZB/WF1 group (41.86 ± 3.14% vs. 45.02 ± 1.22%, p = 0.069) (Fig. 5).

**Figure 6:**
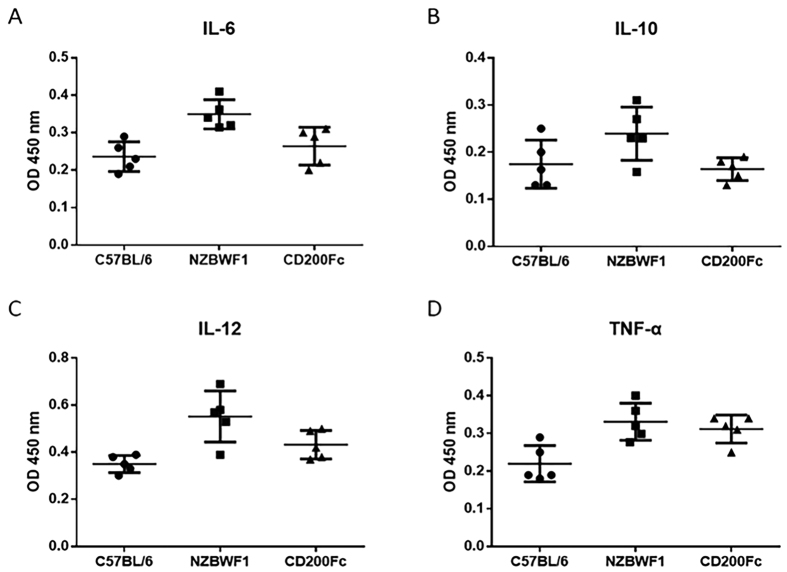
Cytokines produced by dendritic cells in the C57BL/6, NZB/WF1 and CD200-Fc groups. Compared with C57BL/6, the levels of IL-6 (**A**), IL-10 (**B**), (IL-12) and TNF-α (**D**) were higher in the plasma of NZB/WF1 mice, while the levels of IL-6 (**A**) and IL-10 (**B**) in the CD200-Fc group were lower than in the NZB/WF1 group. The means and interquartile ranges are depicted as solid lines.

We finally characterized the effect of CD200-Fc therapy on cytokines produced by DCs after culturing for 24 hours. As shown in Fig. 6, the expression of IL-6, IL-12 and TNF-α was significantly higher in DCs of the NZB/WF1 group compared with those of the C57BL/6 group (0.35 ± 0.05 vs. 0.24 ± 0.04, p = 0.002, 0.55 ± 0.11 vs. 0.35 ± 0.04, p = 0.004 and 0.33 ± 0.05 vs. 0.22 ± 0.05, p = 0.007, respectively), while no significant difference was detected in the levels of IL-10 (0.24 ± 0.06 vs. 0.16 ± 0.05,p = 0.09).When comparing theCD200-Fc group with the NZB/WF1 group, the levels of IL-6 and IL-10 were significantly lower (0.26 ± 0.02 vs. 0.35 ± 0.05, p = 0.017 and 0.21 ± 0.03 vs. 0.24 ± 0.06, p = 0.03, respectively), while no differences in the levels of IL-12 and TNF-α were detected in culture supernatants (0.43 ± 0.06 vs. 0.55 ± 0.11, p = 0.06 and 0.31 ± 0.04 vs. 0.33 ± 0.05, p = 0.51, respectively) (Fig. 6).

## Discussion

It is well recognized that the current therapy for chronic autoimmune diseases, especially SLE, is relatively nonspecific and has limited efficacy. Our previous study demonstrated that CD200 and CD200R1 expression and function are abnormal in SLE and may contribute to the immunologic abnormalities in SLE^16^. In the present study, we conducted new observations by selecting NZB/WF1 mice and examining the therapeutic potential of the recombinant CD200-Fc protein in lupus-prone mice. Our study demonstrated that the percentage of CD200^+^ cells in splenocytes and PBMCs of NZB/WF1 mice was significantly lower than that of C57BL/6 mice. CD200-Fc treatment successfully reduced the levels of anti-dsDNA in the plasma, decreased cytokine production, including IL-6 and IL-10, by DCs and ameliorated renal histopathology. This suggests that CD200 and CD200R1 may play a role in the prevention of SLE and that CD200-Fc is a potential effective therapeutic agent. On the other hand, recent studies revealed that tolerogenic DCs (tolDCs) are special DCs with a promising immunotherapeutic potential for restoring self-tolerance in autoimmune diseases especially SLE^18^,^19^. However, the effects of CD200-Fc on tolDCs remain to be clarified.

Lupus-prone NZB/WF1 mice, which were generated as F1 hybrids between the NZB and NZW strains, have long been used as a classical model of systemic lupus erythematosus. NZB/WF1 mice are characterized by increased levels of autoantibodies, including anti-dsDNA IgG, and glomerulonephritis that becomes apparent at 5–6 months of age^20^. Autoantibodies, especially the most sensitive autoantibodies to dsDNA, play a central role in the onset and development of SLE. The detection of anti-dsDNA autoantibodies improved our ability to diagnose SLE and active disease. Overproduction of autoantibodies and the subsequent hyperactivity of the immune response is believed to be caused by insufficient clearance of apoptotic material by macrophages and DCs^3^. Our findings demonstrated that the level of anti-dsDNA decreased significantly in the CD200-Fc-treatment group, which indicated a role of CD200-Fc in ameliorating the disease activity of SLE.

Lupus nephritis (LN) is one of the most significant causes of morbidity and mortality among patients with SLE, and it is the major cause of death of NZB/WF1 mice^21^. The findings in this study demonstrated that NZB/WF1 mice exhibited glomerular hypercellularity, proliferative glomerulonephritis, higher PAS scores and a higher level of urine protein compared with C57BL/6 mice. The results demonstrated that NZB/WF1 mice developed active LN. For the CD200-Fc group, the histopathology of proliferative glomerulonephritis was ameliorated, with lower PAS scores compared with the NZB/WF1 group. In the study, the difference in urine protein among the NZB/WF1 and CD200-Fc groups was unremarkable, but there appeared to be a decreasing trend after CD200-Fc treatment.

Elevated levels of IL-6 and IL-10 have been associated with modification of the endogenous levels of autoantibodies, including anti-dsDNA, and these increased levels have been shown to be crucial for the development of SLE, whereas blockage of the IL-6 and IL-10 receptors inhibits or delays the onset of renal damage in NZB/WF1 mice^3^,^22^,^23^,^24^. An *in vitro* study demonstrated that incubation of lymphocytes with CD200-Fc inhibits the lymphocyte reaction and alters cytokine production, with decreased production of IL-2 and IFN-gamma and increased production of IL-4 andIL-10^25^. In this study, our findings demonstrated that CD200-Fc reduces the levels of IL-6 and IL-10 produced by DCs. These results may imply that CD200-Fc has a different impact on the production of cytokines, especially IL-10, in different types of cells.

Inappropriate TLR signaling in DCs has been confirmed to be associated with autoimmunity. Up-regulation of TLR4 can disrupt immunological tolerance, which plays a major role in innate immunity and, consequently, induces a lupus-like autoimmune disease in a mouse model and in patients with SLE^26^,^27^. Our study also demonstrated that TLR4 was significantly higher in the DCs of lupus-prone NZB/WF1 mice compared with C57BL/6 mice. However, there was no remarkable difference in the TLR4 expression in the CD200-Fc group compared with the NZB/WF1 group.

In conclusion, we speculate that CD200/CD200R1 might play a general immunosuppressive role and that CD200-Fc may be of value for the treatment of autoimmune disorders caused by dysfunction of DCs in NZB/WF1 mice. These findings are helpful for addressing the increasing interest in exploring the immunotherapeutic feasibility of targeting the CD200/CD200R1 signaling pathway for the management of SLE. To our knowledge, this is the first study to investigate the therapeutic potential of CD200-Fc in SLE, and the findings require further preclinical and clinical exploration.

## Additional Information

**How to cite this article**: Yin, Y. *et al*. Impact of CD200-Fc on dendritic cells in lupus-prone NZB/WF1 mice. *Sci. Rep.* **6**, 31874; doi: 10.1038/srep31874 (2016).

## References

[b1] Koutsokeras T. & Healy T. Systemic lupus erythematosus and lupus nephritis. Nat Rev Drug Discov 13, 173–174, 10.1038/nrd4227 (2014). 24525782

[b2] Sherer Y., Gorstein A., Fritzler M. J. & Shoenfeld Y. Autoantibody explosion in systemic lupus erythematosus: more than 100 different antibodies found in SLE patients. Semin Arthritis Rheum 34, 501–537 (2004). 15505768 10.1016/j.semarthrit.2004.07.002

[b3] Fransen J. H. . The role of dendritic cells in the pathogenesis of systemic lupus erythematosus. Arthritis Res Ther 12, 207, 10.1186/ar2966 (2010). 20423534 PMC2888204

[b4] Shortman K. & Liu Y. J. Mouse and human dendritic cell subtypes. Nat Rev Immunol 2, 151–161, 10.1038/nri746 (2002). 11913066

[b5] Ohnmacht C. . Constitutive ablation of dendritic cells breaks self-tolerance of CD4 T cells and results in spontaneous fatal autoimmunity. J Exp Med 206, 549–559, 10.1084/jem.20082394 (2009). 19237601 PMC2699126

[b6] Banchereau J. & Steinman R. M. Dendritic cells and the control of immunity. Nature 392, 245–252, 10.1038/32588 (1998). 9521319

[b7] Pasare C. & Medzhitov R. Toll pathway-dependent blockade of CD4+CD25+ T cell-mediated suppression by dendritic cells. Science 299, 1033–1036, 10.1126/science.1078231 (2003). 12532024

[b8] Webb M. & Barclay A. N. Localisation of the MRC OX-2 glycoprotein on the surfaces of neurones. J Neurochem 43, 1061–1067 (1984). 6147390 10.1111/j.1471-4159.1984.tb12844.x

[b9] Wright G. J. . Characterization of the CD200 receptor family in mice and humans and their interactions with CD200. J Immunol 171, 3034–3046 (2003). 12960329 10.4049/jimmunol.171.6.3034

[b10] Cherwinski H. M. . The CD200 receptor is a novel and potent regulator of murine and human mast cell function. J Immunol 174, 1348–1356 (2005). 15661892 10.4049/jimmunol.174.3.1348

[b11] Wright G. J. . Lymphoid/neuronal cell surface OX2 glycoprotein recognizes a novel receptor on macrophages implicated in the control of their function. Immunity 13, 233–242 (2000). 10981966 10.1016/s1074-7613(00)00023-6

[b12] Hoek R. M. . Down-regulation of the macrophage lineage through interaction with OX2 (CD200). Science 290, 1768–1771 (2000). 11099416 10.1126/science.290.5497.1768

[b13] Broderick C. . Constitutive retinal CD200 expression regulates resident microglia and activation state of inflammatory cells during experimental autoimmune uveoretinitis. Am J Pathol 161, 1669–1677, 10.1016/s0002-9440(10)64444-6 (2002). 12414514 PMC1850781

[b14] Lyons A., Downer E. J., Costello D. A., Murphy N. & Lynch M. A. Dok2 mediates the CD200Fc attenuation of Abeta-induced changes in glia. J Neuroinflammation 9, 107, 10.1186/1742-2094-9-107 (2012). 22642833 PMC3514341

[b15] Simelyte E. . CD200-Fc, a novel antiarthritic biologic agent that targets proinflammatory cytokine expression in the joints of mice with collagen-induced arthritis. Arthritis Rheum 58, 1038–1043, 10.1002/art.23378 (2008). 18383359

[b16] Li Y. . Aberrant CD200/CD200R1 expression and function in systemic lupus erythematosus contributes to abnormal T-cell responsiveness and dendritic cell activity. Arthritis Res Ther 14, R123, 10.1186/ar3853 (2012). 22621248 PMC3446504

[b17] Kinoshita K. . Retinoic acid reduces autoimmune renal injury and increases survival in NZB/WF1 mice. J Immunol 170, 5793–5798 (2003). 12759464 10.4049/jimmunol.170.11.5793

[b18] Anderson A. E. . LPS activation is required for migratory activity and antigen presentation by tolerogenic dendritic cells. Journal of leukocyte biology 85, 243–250 (2009). 18971286 10.1189/jlb.0608374 PMC2700018

[b19] Llanos C., Mackern-Oberti J. P., Vega F., Jacobelli S. H. & Kalergis A. M. Tolerogenic dendritic cells as a therapy for treating lupus. Clin Immunol 148, 237–245, 10.1016/j.clim.2013.04.017 (2013). 23773922

[b20] Theofilopoulos A. N. & Dixon F. J. Murine models of systemic lupus erythematosus. Adv Immunol 37, 269–390 (1985). 3890479 10.1016/s0065-2776(08)60342-9

[b21] Nozaki Y. . The beneficial effects of treatment with all-trans-retinoic acid plus corticosteroid on autoimmune nephritis in NZB/WF mice. Clin Exp Immunol 139, 74–83, 10.1111/j.1365-2249.2005.02654.x (2005). 15606616 PMC1809273

[b22] Mihara M., Takagi N., Takeda Y. & Ohsugi Y. IL-6 receptor blockage inhibits the onset of autoimmune kidney disease in NZB/WF1 mice. Clin Exp Immunol 112, 397–402 (1998). 9649207 10.1046/j.1365-2249.1998.00612.x PMC1904997

[b23] Ishida H. . Continuous administration of anti-interleukin 10 antibodies delays onset of autoimmunity in NZB/WF1 mice. J Exp Med 179, 305–310 (1994). 8270873 10.1084/jem.179.1.305 PMC2191319

[b24] Rovin B. . A multicenter, randomized, double‐blind, placebo‐controlled study to evaluate the efficacy and safety of treatment with sirukumab (CNTO 136) in patients with active lupus nephritis. Arthritis & Rheumatology (2016). 10.1002/art.39722 PMC5129491 27110697

[b25] Gorczynski R. M. . An immunoadhesin incorporating the molecule OX-2 is a potent immunosuppressant that prolongs allo- and xenograft survival. J Immunol 163, 1654–1660 (1999). 10415071

[b26] Liu B. . TLR4 up-regulation at protein or gene level is pathogenic for lupus-like autoimmune disease. J Immunol 177, 6880–6888 (2006). 17082602 10.4049/jimmunol.177.10.6880

[b27] Dhaouadi T. . Polymorphisms of Toll-like receptor-4 and CD14 in systemic lupus erythematosus and rheumatoid arthritis. Biomark Res 1, 20, 10.1186/2050-7771-1-20 (2013). 24252506 PMC4177616

